# Gastrointestinal Mucormycosis following a *Streptococcus pyogenes* Toxic Shock Syndrome in a Previously Healthy Patient: A Case Report

**DOI:** 10.1155/2012/476719

**Published:** 2012-07-30

**Authors:** Jean-François Roussy, Catherine Allard, Guy St-Germain, Jacques Pépin

**Affiliations:** ^1^Département de Microbiologie et de Maladies Infectieuses, Université de Sherbrooke, CHUS 3001, 12ème Avenue Nord, Sherbrooke, QC, Canada J1H 5N4; ^2^Laboratoire de Santé Publique du Québec, Institut National de Santé Publique du Québec 20045, Chemin Sainte-Marie Sainte-Anne-de-Bellevue, QC, Canada H9X 3R5

## Abstract

Mucormycosis is an uncommon opportunistic infection and the gastrointestinal form is the rarest. *Rhizopus sp*. is the most frequent pathogen and infection occurs almost exclusively in immunocompromised patients. We describe the first case of intestinal mucormycosis occurring after a *Streptococcus pyogenes* toxic shock syndrome in a previously healthy patient caused by *Rhizopus microsporus* var. *azygosporus*.

## 1. Introduction

Mucormycosis is an infection caused by members of the order Mucorales. Relatively uncommon, it is one of the most rapidly fatal infections known to man [1]. The gastrointestinal form is the rarest [1, 2]. Usually, individuals who develop mucormycosis have predisposing risk factors such as diabetes mellitus, deferoxamine chelating therapy, hematological or solid organ malignancies, organ transplantation, neutropenia, and immunosuppressive or corticosteroid therapy [1]. Here, we describe the case of a previously healthy patient who developed a severe form of gastrointestinal mucormycosis a few days after having recovered from a *Streptococcus pyogenes* toxic shock syndrome (TSS). This is to our knowledge the first case of mucormycosis occurring after a *S. pyogenes* TSS.

## 2. Case Report

A previously healthy 53-year-old woman, who had been complaining of respiratory tract symptoms and fever for 3 days, was diagnosed with a *S. pyogenes* necrotizing pneumonia with TSS and transferred to our institution. Upon admission, she was found to have multiorgan failure with acute respiratory distress syndrome and immediately required intubation and vasopressors. She also received IV penicillin, clindamycin, immunoglobulins, activated C-protein, and three days of corticosteroids at physiological dose. Over the following week, the shock resolved and her respiratory parameters improved.

**Figure 1:**
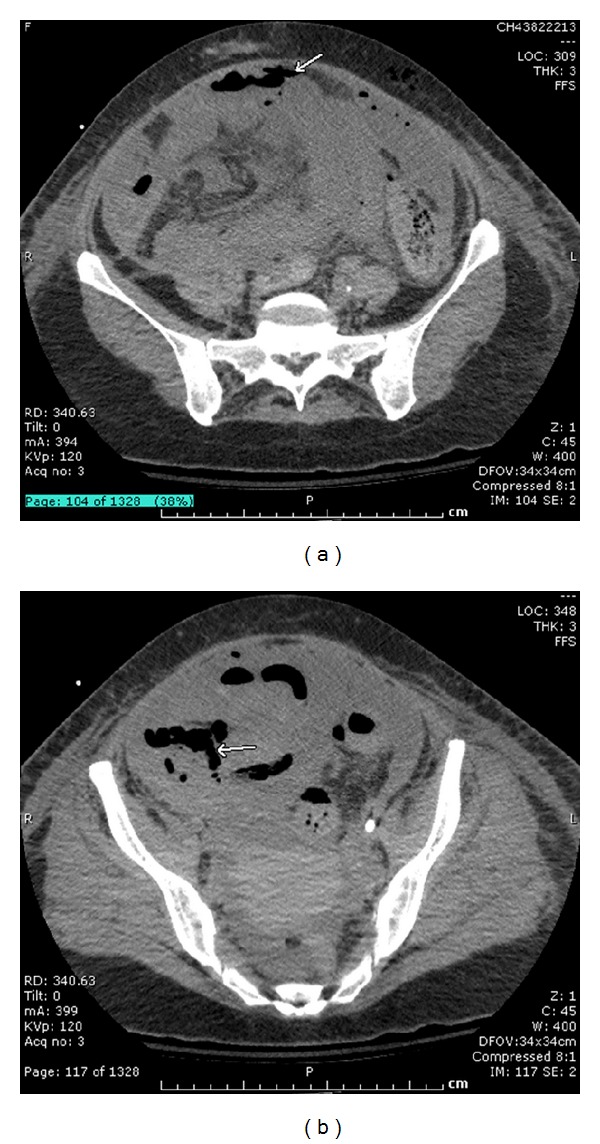
Pneumoperitoneum near the hypogastric region (a); with parietal pneumatosis of the caecum and sigmoid, compatible with acute ischemia (b).

**Figure 2:**
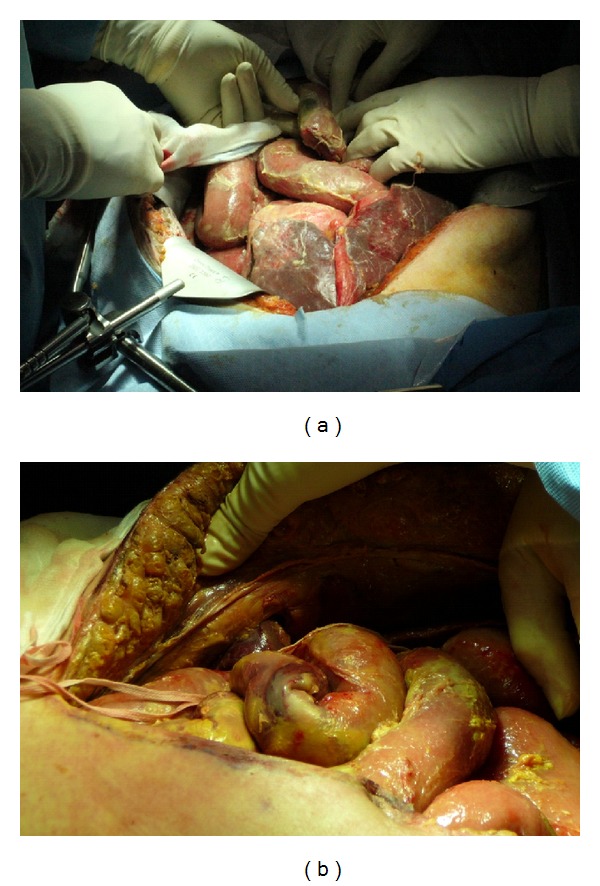
An emergency laparotomy revealed intestinal and colonic necrosis which necessitated a 100 cm resection of the terminal ileum and subtotal colectomy with terminal ileostomy (a). An abundant quantity of nonpurulent but foul-smelling fluid was recovered during the surgery. Thick yellowish membranes covering all intra-abdominal organs were also noticed during surgery (b).

However, on day 10 her condition critically deteriorated, vasopressors were resumed and piperacillin-tazobactam was administered. On physical examination, proctorrhagia and abdominal distension were noted, and she developed leukopenia (Table 1). Abdominal CT scan revealed a pneumoperitoneum near the hypogastric region (Figure 1(a)) with parietal pneumatosis of the caecum and sigmoid, compatible with acute ischemia (Figure 1(b)). An emergency laparotomy revealed intestinal and colonic necrosis which necessitated a 100 cm resection of the terminal ileum and subtotal colectomy with terminal ileostomy (Figure 2(a)). An abundant quantity of nonpurulent but foul-smelling fluid was recovered during the surgery but without any frank fecal peritonitis (Figure 2(b)). Peritoneal fluid cultures were made, and parts of the necrotic small and large bowel were sent to pathology. During the first postoperative day, the septic shock resolved, and blood cultures grew *Bacteroides distasonis*. The following day, a second-look laparotomy confirmed the absence of additional necrosis or ischemia and the wound was closed. The patient remained clinically stable and afebrile, but the leukocytosis progressed. Preliminary results of the peritoneal fluid cultures showed gram-negative rods and hyphae compatible with those of the Mucorales, the latter being initially considered a probable laboratory contaminant given the favorable clinical evolution of the patient at this point.

**Figure 3:**
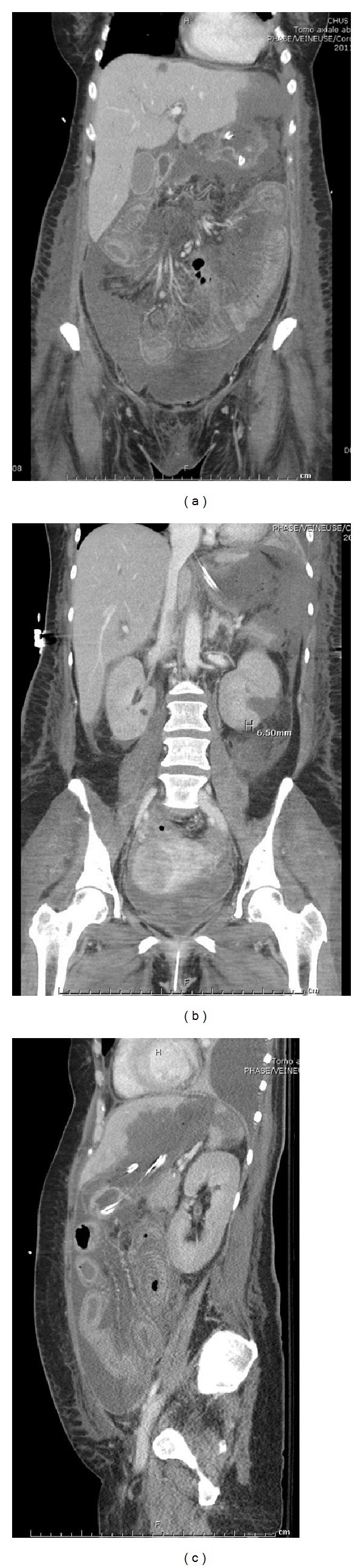
An abdominal CT scan documented a lack of enhancement of the distal portion of the small bowel, including the ileostomy, compatible with ischemia (a). Numerous splenic, hepatic, and renal infarcts were visible (b and c).

**Figure 4:**
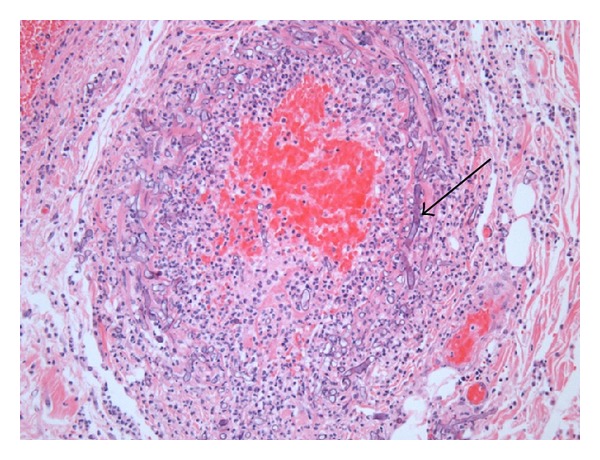
Pathological examination (H&E stain) of the colon showed diffuse active and chronic inflammatory infiltrate with extensive necrosis; broad nonseptate hyphae with irregular branching typical of mucormycosis are present (arrow) within this infiltrate and invade blood vessel walls.

On day 15 (five days postoperatively), the patient became unstable again, with a concomitant rise in lactate and white cell count. With the ileostomy tip and wound borders blackish, a diagnosis of intestinal mucormycosis was suspected. Liposomal amphotericin B was started at 5 mg/kg daily. An abdominal CT scan documented a lack of enhancement of the distal portion of the small bowel, including the ileostomy, compatible with ischemia (Figure 3(a)). Numerous splenic, hepatic, and renal infarcts were visible (Figure 3(b)). Ischemia was suspected on most parts of the residual small bowel. Pathological material sent during the first surgery (appendix, colon, omentum, and intra-abdominal fibrin) all showed broad nonseptate hyphae with irregular branching typical of mucormycosis invading blood vessels (Figure 4). Furthermore, diffuse ischemic necrosis of most parts of the small bowel and colon secondary to the fungus vascular invasion was seen.

**Figure 5:**
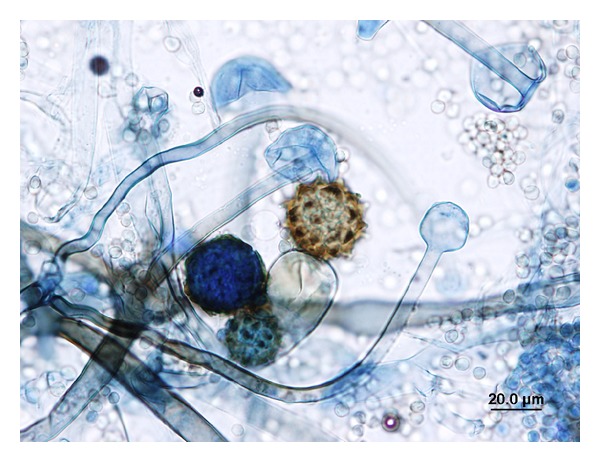
Photomicrograph of sporangiophores and mature azygospores.

A culture of peritoneal fluid on Sabouraud dextrose agar revealed a rapidly growing grayish cottony colony following incubation at 30°C for 3 days. The organism was thermophilic, displaying good growth at 45°C. Microscopic examination revealed large and mostly nonseptate hyphae, rhizoids, short unbranched and brown sporangiophores up to 500 *μ*m in length, spherical sporangia, ovoidal sporangiospores 4–7 *μ*m long, occasional chlamydospores, and numerous pale to dark brown azygospores (Figure 5). From these features the fungus was identified as *Rhizopus microsporus* var. *azygosporus.* An DNA sequence obtained for the complete ITS1-5.8S-ITS 2 region revealed 100% homology with numerous *R. microsporus* strains in GenBank, including *R. azygosporus* strains CBS 357.93 and CBS 357.92 (GenBank accession numbers DQ119008 and AB097391). *R. azygosporus* is now considered a variety within *R. microsporus*. However, the ITS sequence does not allow discrimination of the varieties which are still assigned by morphology. The isolate was deposited at the Laboratoire de santé publique du Québec under accession number LSPQ-00966. The in vitro susceptibility of the isolate to antifungal drugs was determined using the Clinical and Laboratory Standards Institute's broth microdilution method M38-A2 [3]. The results were as follows: amphotericin B, 1 mg/L; 5-fluorocytosine >64 mg/L, itraconazole, 1 mg/L; posaconazole, 0.5 mg/L; voriconazole, >8 mg/L; anidulafungin, >8 mg/L; caspofungin, >8 mg/L; micafungin, >8 mg/L.

 Micafungin was added to the therapeutic regimen because of a possible synergistic effect with amphotericin B against mucoraceous fungi. Daily laparotomies with broad resections of the necrotic tissues were performed. An immunology consultation did not establish an immune deficit, but immunoglobulins and interferon-gamma were administered nonetheless to optimize the phagocytic function of leucocytes.

Unfortunately, even after eight interventions (including multiple liver segment resections, partial gastrectomy, splenectomy, multiple intestinal resections, salpingectomy, left radical nephrectomy, distal pancreatectomy, and left diaphragm resection), mucormycosis continued to progress relentlessly over surgical margins. On day 27, it was decided to cease treatments. The patient died a few hours later.

## 3. Discussion

The main protagonists of host defense mechanisms in mucormycosis are mononuclear and polymorphonuclear phagocytes, both of which are impaired in most of the clinical settings associated to the previously mentioned risk factors. In the normal host, phagocytes kill the fungus by generating oxidative metabolites and cationic peptides defensins [2]. In vitro and in vivo studies have shown that corticosteroids also impede the capacity of macrophages to prevent germination of sporangiospores [4]. Iron is an essential element in the development of mucormycosis. To combat this infection, the normal host uses specialized iron-binding proteins to sequester iron in serum [1].

 Clinical presentation depends on the predisposing conditions and the route of infection, resulting in either cutaneous, rhinocerebral, pulmonary, gastrointestinal (GI), or disseminated mucormycosis [5]. Angioinvasion and hematogenous dissemination to other organs are the hallmarks of mucormycosis. Gl mucormycosis is the rarest form and involves mainly the stomach, the colon, and the small bowel [6]. The fungus invades the bowel wall and blood vessels, leading to bowel ischemia, necrosis, perforation, peritonitis, or massive hemorrhage [5, 7–9].

A recent study of 190 isolates of molecularly identified *Mucorales* species from clinical specimens in the United States revealed that 22% were *R. microspores* [10]. None were isolated from gastrointestinal specimens and none were morphologically identified as *R. microsporus* var. *azygosporus*. This organism has been very rarely implicated in human disease. To our knowledge, this case is the fifth described in the literature*. R. microsporus* var. *azygosporus* was previously reported as the causative agent of three fatal cases of gastrointestinal infection in premature babies [11] and in one case of deadly cutaneous mucormycosis in a 54-year-old woman with systemic lupus erythematosus receiving intense immunosuppressive therapy [12].

Our case is intriguing because this patient developed intestinal mucormycosis despite the lack of any immunosuppression prior to streptococcal TSS and of any of the other classical predisposing factors. She was HIV-negative, and no cellular or humoral defects were elicited. It is unlikely that the short course of corticosteroids impaired her immune response. Consequently, could the streptococcal TSS have predisposed her into developing this rare form of mucormycosis?

Patients with diabetic ketoacidosis are at high risk of developing rhinocerebral mucormycosis [2, 13] because of the neutrophil dysfunction induced by this condition and also possibly because acidotic patients have elevated levels of available serum iron due to a temporary impairment of iron-binding transferrin [14]. Our patient had experienced severe metabolic acidosis for several days (Table 1), and we speculate that the infection may have been precipitated to some extent by an elevation in the amount of serum iron accessible to the fungus.

Streptococcal pyrogenic exotoxins cause a transient decrease in RNA synthesis in Kupffer cells, which in turn alters the reticuloendothelial system function [15]. In our patient, this may have ultimately impaired the phagocytosis of fungal elements. West et al. proposed that in the presence of concomitant bacterial and fungal infection/colonization, once the bacteria are killed following an antimicrobial therapy, there may be a progression of the mucormycosis due to the absence of bacteria competing for nutrients [16]. Furthermore, endothelial damages caused by the TSS-related cytokines storm may have facilitated the fungus' penetration of blood vessels.

It remains unclear how the fungal pathogen was introduced into her GI tract. This is generally attributed to the ingestion of contaminated foods or the use of contaminated instruments [17]. For example, hospital-acquired infections due to *Rhizopus spp*. have been described following the use of wooden tongue depressors [18]. Similarly, gastric mycoses have developed after nasogastric intubation or other conditions leading to the development of gastric or colonic ulcers, suggesting that an initial ulceration may facilitate fungal entry [17]. Environmental cultures (air, surfaces, wooden tongue depressors, nasogastric tubes, bandages, and mechanical ventilators) in our intensive care unit did not reveal the presence of *R. microsporus*. Moreover, during the subsequent year, no other case of mucormycosis was identified in our center, and no *R. microsporus* was isolated in any clinical specimen. As fungi of the order Mucorales are ubiquitous in the environment, it seems likely that she was colonized with the fungus prior to her hospital admission and became infected after multiple invasive procedures.

Because of its vascular tropism, rapid diagnosis of mucormycosis is essential to lower mortality [7], which can be higher than 50% in patients with hematological malignancies [6]. Unfortunately, diagnosis is difficult and requires a biopsy of the organ(s) involved. For example, antemortem diagnosis was made in only 9% of 185 cases of disseminated mucormycosis [19].

Imaging findings of GI mucormycosis have rarely been reported in the radiological literature. In early-stage GI mucormycosis, CT may show nonspecific bowel wall thickening, with or without target sign, and decreased bowel wall enhancement, representing bowel ischemia. When the involved bowel segment becomes frankly necrotic, the bowel wall is thinned or even disappears, as in our case [6]. Timely presumptive radiological diagnosis of mucormycosis could improve the prognosis. In the proper clinical setting, CT findings of a long segmental discontinuity of bowel wall along with a large amount of hematoma and localized peritonitis may be suggestive of extensive infiltration of angioinvasive fungi, although definite diagnosis can be made only by histological examinations [6].

Successful treatment requires a combination of aggressive surgical removal of necrotic tissues along with long-term administration of intravenous antifungal therapy. A recent study showed that susceptibility of Mucorales to antifungals differs according to family, genus, and species [20]. The most active drugs in vitro remain amphotericin B and posaconazole, the latter being less useful in the acute setting because of its oral formulation. To date, the drug of choice has been amphotericin B which, combined with surgical intervention, can enhance survival [5]. Recent studies have suggested that the combination of amphotericin B with caspofungin increases survival compared to amphotericin B lipid complex monotherapy [13, 21, 22]. In our case, extensive surgery and polyene-echinocandin combination therapy failed to halt the spread of the pathogen.

 In summary, this is to our knowledge the first case of intestinal mucormycosis occurring after a *S. pyogenes* TSS. The diagnosis of mucormycosis is rarely suspected in patients without any known risk factor and isolation of a mucoraceous fungus in these circumstances is often considered nonsignificant. This delay in diagnosis will further worsen the prognosis. TSS may induce a state of reticuloendothelial dysfunction and altered iron metabolism which predisposes to mucormycosis.

## Figures and Tables

**Table 1:** Laboratory data.

Variable	Reference range, adults	On admission	Day 1	Day 2	Day 7	Day 10 (surgery number 1)
Hemoglobin (g/dL)	12.0–16.0 (women)	9.4	8.9	10.0	10.3	8.6
White cell count (per mm^3^)	4500–11,000	49,000	50,000	46,200	37,000	12,600
Lactate (mmol/L)	0.5–2.2	5.06	4.98	4.78	1.70	2.25
Creatinine (mg/dL)	0.60–1.50	2.73	1.27	0.89	2.52	1.80
pH	7.35–7.45	7.26	7.24	7.10	7.53	7.48
HCO_3_ (mmol/L)	22–26	13.2	22.6	20.6	29.9	24.6

## References

[1] Spellberg B, Edwards J, Ibrahim A (2005). Novel perspectives on mucormycosis: pathophysiology, presentation, and management. *Clinical Microbiology Reviews*.

[2] Ribes J, Vanover-Sams C, Baker DJ (2000). Zygomycetes in human disease. *Clinical Microbiology Reviews*.

[3] CLSI (2009). Reference method for broth dilution antifungal susceptibility testing of filamentous fungi. Approved standard. *CLSI Document*.

[4] Waldorf AR, Ruderman N, Diamond RD (1984). Specific susceptibility to mucormycosis in murine diabetes and bronchoalveolar macrophage defense against *Rhizopus*. *Journal of Clinical Investigation*.

[5] Kauffman CA, Malani AN (2007). Zygomycosis: an emerging fungal infection with new options for management. *Current Infectious Disease Reports*.

[6] Kim HJ, Rha SE, Kang WK (2011). A patient with neutropenic fever and abdominal pain showing absent bowel wall on CT. *British Journal of Radiology*.

[7] Hyvernat H, Dunais B, Burel-Vandenbos F, Guidicelli S, Bernardin G, Gari-Toussaint M (2010). Fatal peritonitis caused by *Rhizopus microsporus*. *Medical Mycology*.

[8] Nayak S, Satish R, Gokulnath, Savio J, Rajalakshmi T (2007). Peritoneal mucormycosis in a patient on CAPD. *Peritoneal Dialysis International*.

[9] Polo JR, Luño J, Menarguez C, Gallego E, Robles R, Hernandez P (1989). Peritoneal mucormycosis in a patient receiving continuous ambulatory peritoneal dialysis. *American Journal of Kidney Diseases*.

[10] Alvarez E, Sutton DA, Cano J (2009). Spectrum of zygomycete species identified in clinically significant specimens in the United States. *Journal of Clinical Microbiology*.

[11] Schipper MA, Maslen MM, Hogg GG, Chow CW, Samson RA (1996). Human infection by *Rhizopus azygosporus* and the occurrence of azygospores in Zygomycetes. *Journal of Medical and Veterinary Mycology*.

[12] Fujimoto A, Nagao K, Tanaka K, Yamagami J, Udagawa SI, Sugiura M (2005). The first case of cutaneous mucormycosis caused by *Rhizopus azygosporus*. *British Journal of Dermatology*.

[13] Walsh TJ, Kontoyiannis DP (2008). What is the role of combination therapy in management of zygomycosis?. *Clinical Infectious Diseases*.

[14] Artis WM, Fountain JA, Delcher HK, Jones HE (1982). A mechanism of susceptibility to mucormycosis in diabetic ketoacidosis: transferrin and iron availability. *Diabetes*.

[15] Schlievert PM, Bettin KM, Watson DW (1980). Inhibition of ribonucleic acid synthesis by group A *streptococcal pyrogenic exotoxin*. *Infection and Immunity*.

[16] West BC, Oberle AD, Kwon-Chung KJ (1995). Mucormycosis caused by *Rhizopus microsporus var. microsporus*: cellulitis in the leg of a diabetic patient cured by amputation. *Journal of Clinical Microbiology*.

[17] Antoniadou A (2009). Outbreaks of zygomycosis in hospitals. *Clinical Microbiology and Infection*.

[18] Maraví-Poma E, Rodríguez-Tudela JL, de Jalón JG (2004). Outbreak of gastric mucormycosis associated with the use of wooden tongue depressors in critically ill patients. *Intensive Care Medicine*.

[19] Chakrabarti A, Das A, Sharma A (2001). Ten years’ experience in zygomycosis at a tertiary care centre in India. *Journal of Infection*.

[20] Vitale RG, de Hoog GS, Schwarz P, Dannaoui E, Deng S (2012). Antifungal susceptibility and phylogeny of opportunistic members of mucorales. *Journal of Clinical Microbiology*.

[21] Rodriguez MM, Serena C, Marine M, Pastor FJ, Guarro J (2008). Posaconazole combined with amphotericin B, an effective therapy for a murine disseminated infection caused by *Rhizopus oryzae*. *Antimicrobial Agents and Chemotherapy*.

[22] Reed C, Bryant R, Ibrahim AS (2008). Combination polyene-caspofungin treatment of rhino-orbital-cerebral mucormycosis. *Clinical Infectious Diseases*.

